# Evaluating Word Representation Features in Biomedical Named Entity Recognition Tasks

**DOI:** 10.1155/2014/240403

**Published:** 2014-03-06

**Authors:** Buzhou Tang, Hongxin Cao, Xiaolong Wang, Qingcai Chen, Hua Xu

**Affiliations:** ^1^Department of Computer Science, Harbin Institute of Technology Shenzhen Graduate School, Shenzhen, Guangdong 518055, China; ^2^School of Biomedical Informatics, The University of Texas Health Science Center at Houston, Houston, TX 77030, USA; ^3^Department of Medical Informatics, Second Military Medical University, Shanghai 200433, China

## Abstract

Biomedical Named Entity Recognition (BNER), which extracts important entities such as genes and proteins, is a crucial step of natural language processing in the biomedical domain. Various machine learning-based approaches have been applied to BNER tasks and showed good performance. In this paper, we systematically investigated three different types of word representation (WR) features for BNER, including clustering-based representation, distributional representation, and word embeddings. We selected one algorithm from each of the three types of WR features and applied them to the JNLPBA and BioCreAtIvE II BNER tasks. Our results showed that all the three WR algorithms were beneficial to machine learning-based BNER systems. Moreover, combining these different types of WR features further improved BNER performance, indicating that they are complementary to each other. By combining all the three types of WR features, the improvements in *F*-measure on the BioCreAtIvE II GM and JNLPBA corpora were 3.75% and 1.39%, respectively, when compared with the systems using baseline features. To the best of our knowledge, this is the first study to systematically evaluate the effect of three different types of WR features for BNER tasks.

## 1. Introduction

Biomedical Named Entity Recognition (BNER), which extracts important biomedical concepts such as genes and proteins, is a crucial step of natural language processing (NLP) in the biomedical domain. Because of the complexity of biomedical nomenclature, BNER has been a challenging task. First, the same biomedical named entities can be expressed in various forms. For example, gene names often contain alphabets, digits, hyphens, and other characters, thus having many variants (e.g., “HIV-1 enhancer” versus “HIV 1 enhancer”). Moreover, many abbreviations (e.g., “IL2” for “Interleukin 2”) have been used for biomedical named entities. Sometimes, the same entity can have very different aliases (e.g., “PTEN” and “MMAC1” refer to the same gene) [1]. Another challenge of BNER is the ambiguity problem. The same word or phrase can refer to more than one type of entities or does not refer to an entity depending on context (e.g., “TNF alpha” can refer to a protein or DNA). All these phenomena make the named entity recognition (NER) task in the biomedical domain more difficult than that in open domains such as newswire.

Considerable efforts have been devoted to BNER research, including some shared-task challenges, such as JNLPBA (Joint Workshop on Natural Language Processing in Biomedicine and its Applications) in 2004 [2] and BioCreAtIvE (Critical Assessment for Information Extraction in Biology Challenge) II GM (gene mention) in 2007 [3]. Different methods have been developed for BNER, mainly falling into three categories: (1) dictionary-based methods [4]; (2) rule-based methods [5, 6]; and (3) machine learning-based approaches [7]. Among them, machine learning-based methods have demonstrated their advantage and showed better performance than the other two categories of methods when a large annotated corpus is available. For example, all the systems in the JNLPBA challenge used one or more machine learning algorithms and greatly outperformed the dictionary-based baseline system [2].

Various machine learning algorithms have been used in BNER, including hidden Markov models (HMM) [8, 9], maximum entropy Markov models (MEMM) [10, 11], conditional random fields (CRF) [12, 13], and support vector machines (SVM) [14, 15]. Among them, CRF have been recognized as a reliable, high-performance algorithm for different BNER-shared tasks [12, 16, 17]. Another important aspect for machine learning-based BNER approaches is features used for building the classification models. Current BNER systems often use different types of linguistic features including morphological, syntactic, semantic information of words, and domain-specific features from biomedical terminologies such as BioThesaurus [18] and UMLS (Unified Medical Language System) [19]. More recently, there is an interest in using new features from unlabeled corpora to improve machine learning-based NER systems. One of the most representative techniques is word representation (WR) [20], which uses unsupervised learning algorithms to generate word-level back-off features from an unlabeled corpus. Those WR features could contain latent syntactic/semantic information of a word. Currently, only very few studies have applied WR features to BNER tasks. For example, Kuksa and Qi investigated the effect of distributed WR features for BNER and their evaluation using BioCreativeII GM corpus showed a significant improvement when adding these features [21].

A large number of techniques have been proposed to extract WR features, such as hyperspace analogue to language (HAL) [22], LSA (latent semantic analysis) [23], latent Dirichlet allocation (LDA) [24], random indexing (RI) [25], canonical correlation analysis (CCA) [26], Brown clustering [27], and neural language models [28–32]. According to a review by Turian et al. [20], WR features can be divided into three categories: (1) clustering-based methods such as Brown clustering [27]; (2) distributional representations, such as LSA [23], LDA [24], and random indexing [25]; and (3) word embeddings (also called distributed representations), such as neural language models [28]. Recently, WR techniques have been widely used to improve various machine learning-based NLP tasks, such as part-of-speech (POS), chunking, and NER in newswire domain [20], and entity recognition in clinical text [33–35]. Word embeddings have also been applied to the biomedical domain and showed improvement on entity recognition in biomedical literature [21]. Nevertheless, the contribution of different types of WR features to BNER has not been extensively investigated yet.

The goal of this study is to systematically evaluate three types of WR features, as well as their combinations, on BNER tasks. We selected one algorithm from each of the three types of WR features and applied them to the JNLPBA and BioCreAtIvE II BNER tasks. Our results showed that all the three WR algorithms were beneficial to machine learning-based BNER systems. Moreover, these different WR features were also complementary to each other. By combining all the three types of WR features, the improvements in* F*-measure on the BioCreAtIvE II GM and JNLPBA corpora were 3.75% and 1.39%, respectively, when compared with the systems using baseline features. To the best of our knowledge, this is the first study to systematically evaluate the effect of three different types of WR features for BNER tasks.

## 2. Materials and Methods

### 2.1. Data Sets

Our experiments were conducted on the BioCreAtIvE II GM corpus and JNLPBA corpus. The BioCreAtIvE II GM corpus consists of 20,000 sentences (15,000 sentences for training and 5,000 sentences for test) from MEDLINE citations, where gene/protein names were manually annotated. The JNLPBA corpus consists of 22,402 sentences from MEDLINE (18,546 sentences for training and 3,856 for test), where five categories of entities (protein, DNA, RNA, cell line, and cell type) were manually annotated.  Table 1 shows the counts of different types of entities in two corpora. Sentences are pretokenized in the JNLPBA but not in the BioCreAtIvE II GM corpus. In our experiments, we used GENIA tagger (http://www.nactem.ac.uk/GENIA/tagger/) to perform tokenization for the BioCreAtIvE II GM corpus.

### 2.2. Machine Learning Algorithm

Given the tokenized text, the NER task can be modeled as a sequence labeling problem by assigning each token to a label to determinate the boundaries of named entities, such as B = beginning of an entity, I = inside an entity, and O = outside of an entity (see examples in Table 2). In this study, we used conditional random fields (CRF), a probabilistic undirected graphical model, for two BNER tasks. CRF have been widely used in NER tasks in various domains including biomedicine and have shown the state-of-the-art performance. For example, almost all top-ranked teams in BioCreAtIvE II GM and JNLPBA challenges utilized CRF [2, 3].

### 2.3. Features

In this study, we included four types of features: one set of basic features such as bag-of-word and part-of-speech (POS) and three types of WR features. Although any unlabeled MEDLINE corpus can be used to generate WR features, in this study, we treated the BioCreAtIvE II GM and JNLPBA corpora as unlabeled collections to generate WR features. Details of each type of features are described as follows.

#### 2.3.1. Basic Features

Basic features include stemmed words in a context window of [−2, 2], including unigrams, bigrams, and trigrams. Porter stemming algorithm was used to extract the stem of each normalized word. In addition, we also added part-of-speech (POS) tags of words in the same window as features. POS tagging was done by GENIA tagger (http://www.nactem.ac.uk/GENIA/tagger/).

#### 2.3.2. Clustering-Based WR

**Figure 1:**
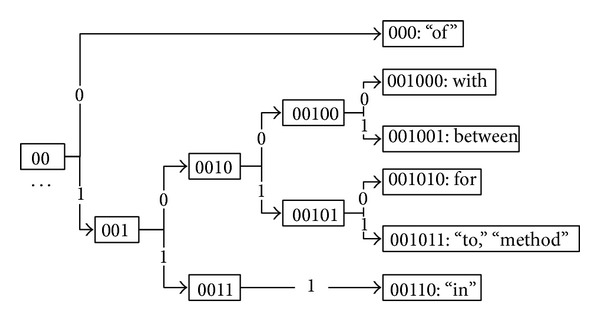
A hierarchical structure fragment generated by Brown clustering for 7 words from the JNLPBA corpus.

The clustering-based WR induces clusters over words in an unlabeled corpus and represents a word by cluster(s) it belongs to. The idea is that words that are semantically/syntactically similar tend to be in the same or close clusters. Similar to [34], we adopted the Brown clustering algorithm [27] (https://github.com/percyliang/brown-cluster/), a hierarchical clustering algorithm. We ran the Brown clustering algorithm and generated hierarchical clusters of all the words in each corpus, represented by a binary tree, whose leaf nodes are all the words.  Figure 1 shows a fragment of a hierarchical cluster containing 7 words from the JNLPBA corpus. The numbers in the squares (e.g., 00) represent the subpaths starting from the root of the cluster encoded with a binary sequence, and words that share more similar subpaths are semantically closer. In our experiments, all subpaths from the root to a word (i.e., a leaf node) were used as its features. For example, the following features were extracted for the word “for” (001010): {“0,” “00,” “001,” “0010,” “00101,” and “001010”}. The number of clusters for running Brown clustering algorithm was selected from the set of {50, 100, 200, 500, 1000, and 2000}. The optimized cluster numbers were 500 and 200 on the BioCreAtIvE II GM and JNLPBA corpora, respectively.

#### 2.3.3. Distributional WR

The distributional WR is a word cooccurrence-based approach to latent semantics, which uses statistical approximations to reduce a word cooccurrence matrix of high dimensionality to a latent semantic matrix of low dimensionality. Then, a semantic thesaurus can be constructed from the semantic matrix by computing similarities of each word pair or clusters by clustering algorithms. Finally, a word can be represented by other words in the semantic thesaurus or cluster(s) it belongs to. In this study, we reduced dimension of cooccurrence matrix using random indexing [25] and then built a semantic thesaurus using cosine function for semantic similarity computing. Finally, a word was represented by its nearest semantic words (with similarity) in the semantic thesaurus.  Table 3 shows a fragment of the semantic thesaurus of 3 words in the JNLPBA corpus. The word in the first row of each column (e.g., “zymosan-tr”) is a word in the corpus, and other words in the same column (e.g., “interferon-tr”) are words in the semantic thesaurus, sorted by semantic similarity score (e.g., “0.276595744681”). In our experiments, each word was represented by *N*-nearest semantic words, where *N* was selected from the set of {5, 10, 20, and 50}. The optimized *N*s were 10 and 50 on the BioCreAtIvE II GM and JNLPBA corpora, respectively. For example, the following features were extracted for the word “zymosan-tr”: {“interferon-tr”: 0.276595744681, “jak-1-defici”: 0.243902439024, “p388”: 0.236842105263, “ald-induc”: 0.228571428571, and “alpha-prolif”: 0.22}.

#### 2.3.4. Word Embeddings

Word embeddings (also called distributed word representations) induce a real valued latent syntactic/semantic vector for each word from large unlabeled corpus by continuous space language models. A word can be directly represented by its vector and similar words are likely to have similar vectors. In our experiments, we adopted the method in [32] (https://code.google.com/p/word2vec/), a neural network language model to generate word embeddings (shown in Table 4). The dimension of each word vector was selected from the set of {50, 100, 200, and 300}. The optimized dimensions of each word vector were 50 and 100 on the BioCreAtIvE II GM and JNLPBA corpora, respectively.

### 2.4. Experiments and Evaluation

In this study, we started with a baseline system that adopted basic features such as bag-of-word and POS mentioned in the previous section. Then, we evaluated the effect of three types of WR features: clustering-based, distributional word representations, and word embeddings, by adding each of them individually to the baseline system. Furthermore, we evaluated different combinations of three types of WR features. All WR features were derived from the entire unlabeled corpora of BioCreAtIvE II GM and JNLPBA.

We used CRFsuite (http://www.chokkan.org/software/crfsuite/) as an implementation of CRF and optimized its parameters on the training set of each corpus by 10-fold crossvalidation. The optimum number for each type of WR features was also determined during 10-fold crossvalidation. The performance of different approaches was evaluated using the test set of each corpus and reported as standard precision, recall and *F*-measure, calculated using the official evaluation tool provided by the organizers of the two challenges [2, 3].

## 3. Results

Table 5 shows the performance of CRF-based BNER approaches on the test sets of BioCreAtIvE II GM and JNLPBA corpora, when three different types of WR features were added individually or in combination. As shown in the table, each individual type of WR features improved the performance of BNER systems. When the clustering-based, distributional, and word embedding WR features were individually added to the basic features, the *F*-measures were improved by 2.1%, 2.86%, and 1.53% on the BioCreAtIvE II GM corpus and by 1.2%, 0.55%, and 0.49% on the JNLPBA corpus, respectively. Different types of WR features seemed to be complementary to each other. BNER systems with any two types of WR features outperformed these with a single type of WR features. For example, when both clustering-based and distributional WR features were used, the *F*-measures were improved by 3.38% on the BioCreAtIvE II GM corpus (versus improvements of 2.1% and 2.86% when either clustering-based or distributional WR features were added to the baseline) and 1.38% on the JNLPBA corpus (versus improvements of 1.2% and 0.55% when either clustering-based or distributional WR features were individually added to the baseline). When all three types of WR features were used, the BNER systems achieved the best performance on both the BioCreAtIvE II GM and JNLPBA corpora, with the highest *F*-measures of 80.96% and 71.39% (improvements of 3.75% and 1.39% compared to the baseline), respectively.

## 4. Discussion

In this paper, we investigated the effect of three types of WR features, including clustering-based representation, distributional representation, and word embeddings, on machine learning-based BNER systems. Evaluation on both the BioCreAtIvE II GM and JNLPBA corpora showed that each type of WR features was beneficial to the CRF-based BNER systems, with an *F*-measure improvement ranging from 0.49% to 2.86%. Moreover, our results also demonstrated that combining different types of WR features further improved BNER performance, indicating that these different types of WR features were complementary to each other. All these findings provide valuable insight into efficient use of WR features in BNER tasks.

Another interesting finding is that the improvements by different WR features varied among different corpora. For example, the distributional WR features achieved the highest improvement on the BioCreAtIvE II GM corpus (i.e., 2.86% in *F*-measure), while it was the clustering-based features that achieved the highest improvement on the JNLPBA corpus (i.e., 1.2% in *F*-measure). We also noticed that the performance gain by WR features was mainly from higher recalls, because unsupervised word representation features could help detect more entities that do not appear in the training data set. For example, the “Baseline+WR1+WR2+WR3” system detected additional 476 entities (288 entities were correct) on the JNLPBA corpus, when compared with the “Baseline” system.

To compare our system with other state-of-the-art BNER systems, we further included additional features to our best systems, including word shape, prefixes, suffixes, orthographic features, and morphological features, all of which were widely used in previously developed BNER systems [9]. The best *F*-measures with all the features were 85.83% and 72.74% on the BioCreAtIvE II GM and JNLPBA corpora, respectively. As expected, WR features were still helpful, though the improvements by WR features were much less (0.2% and 0.3% *F*-measures, resp.) when all other features were used. Anyway, these results are competitive; for example, the *F*-measure on the JNLPBA corpus (72.74%) was higher than the best system in the JNLPBA 2004 challenge. However, our system's performance on BioCreAtIvE II GM was still not as good as others such as [3, 18, 36, 37]. The main reason is that those systems used extensive domain knowledge, ensemble approaches, or postprocessing modules. We believe that adding WR features to these existing systems would further improve their performance.

This study has limitations. For each type of WR features, only one algorithm was implemented and evaluated. It is worth investigating other algorithms in each type of WR features, which is one of our future works. In addition, we treated the annotated corpora as unlabeled data sets to generate WR features. In reality, we could generate WR features from a much larger unlabeled corpus such as MEDLINE, which may achieve even higher performance.

## 5. Conclusions

In this study, we investigated the use of three different types of WR features in biomedical entity recognition. Our evaluation on the BioCreAtIvE II GM and JNLPBA corpora showed that not only individual types of WR features were beneficial to BNER tasks but also different types of WR features could be combined and further improve the performance of BNER systems.

## Figures and Tables

**Table 1:** Counts of different types of entities in two corpora used in this study.

Corpus	BioCreAtIvE II GM		JNLPBA					
	Gene/protein	Total	Protein	DNA	RNA	Cell line	Cell type	Total
Training	18,265	18,265	30,269	9,534	951	3,830	6,718	51,301
Test	6,331	6,331	5,067	1,056	118	500	1,921	8,662

**Table 2:** Examples of named entities represented by BIO labels. The first sentence comes from the JNLPBA corpus and the second sentence comes from the BioCreAtIvE II GM corpus.

Example 1	Token	IL-2	gene	expression	and	NF-kappa	B	activation	*⋯*
	Label	B-DNA	I-DNA	O	O	B-protein	I-protein	O	*⋯*

Example 2	Token	Comparison	with	alkaline	phosphatases	and	5	—	nucleotidase
	Label	O	O	B-GM	I-GM	O	B-GM	I-GM	I-GM

**Table 3:** A fragment of the semantic thesaurus of 3 words in the JNLPBA corpus, after running random indexing.

zymosan-tr	zymogen	ym268
interferon-tr: 0.276595744681 jak-l-defici: 0.243902439024 p388: 0.236842105263 ald-induc: 0.228571428571 alpha-prolif: 0.22 *⋯*	monocyte/b-cell-specif: 0.359477124183 tubulointerstitium: 0.314720812183 c-fms: 0.284768211921 simplest: 0.282608695652 isotype-specif: 0.277777777778 *⋯*	jak-l: 0.272425249169 forskolin: 0.272388059701 nf-a 1: 0.265560165975 icp0: 0.261467889908 betal: 0.25 *⋯*

**Table 4:** Word embeddings of 4 words in the JNLPBA corpus. Each number denotes the feature value in a latent semantic/syntactic space.

the: 0.067476 −0.017934 0.036855 0.348073 0.063362 −0.138005 −0.144527 −0.014324 0.161269 0.152643 …
of: 0.067905 −0.074922 0.012121 0.050542 0.327945 0.098191 −0.087244 0.194758 0.218592 −0.115941 …
gene: −0.254542 0.100417 −0.124032 0.084818 −0.279409 0.081752 −0.378949 −0.068434 −0.050847 0.142284 …
transcript: −0.157966 −0.303626 0.010010 −0.081133 −0.111763 −0.088829 −0.160671 0.185505 0.097515 −0.014036 …

**Table 5:** Performance of CRF-based BNER systems when different types of WR features were used.

System	BioCreAtIvE II GM (%)			JNLPBA (%)		
	Precision	Recall	*F*-measure	Precision	Recall	*F*-measure
Baseline	87.31	69.20	77.21	71.37	68.68	70.00
Baseline + WR1	86.55	73.18	79.31	70.96	71.44	71.20
Baseline + WR2	87.34	73.91	80.07	71.59	69.55	70.55
Baseline + WR3	86.56	72.22	78.74	71.11	69.88	70.49
Baseline + WR1 + WR2	86.56	75.39	80.59	70.99	71.77	71.38
Baseline + WR1 + WR3	85.77	74.65	79.82	70.77	71.87	71.31
Baseline + WR2 + WR3	87.03	74.90	80.51	71.19	70.41	70.80
Baseline + WR1 + WR2 + WR3	86.54	76.05	80.96	70.78	72.00	71.39

*WR1, WR2, and WR3 denote three different types of word representation features: clustering-based, distributional, and word embeddings features, respectively.

## References

[1] Leser U, Hakenberg J (2005). What makes a gene name? Named entity recognition in the biomedical literature. *Briefings in Bioinformatics*.

[2] Kim J-D, Ohta T, Tsuruoka Y, Tateisi Y, Collier N Introduction to the bio-entity recognition task at JNLPBA.

[3] Smith L, Tanabe LK, Ando R (2008). Overview of BioCreative II gene mention recognition. *Genome Biology*.

[4] Gaizauskas R, Demetriou G, Humphreys K Term Recognition and Classification in Biological Science Journal Articles.

[5] Fukuda K, Tamura A, Tsunoda T, Takagi T (1998). Toward information extraction: identifying protein names from biological papers. *Pacific Symposium on Biocomputing. Pacific Symposium on Biocomputing*.

[6] Proux D, Rechenmann F, Julliard L, Pillet VV, Jacq B (1998). Detecting gene symbols and names in biological texts: a first step toward pertinent information extraction. *Genome Informatics Work. Genome Informatics*.

[7] Nobata C, Collier N, Tsujii J Automatic term identification and classification in biology texts.

[8] Rabiner LR (1989). A tutorial on hidden markov models and selected applications in speech recognition. *Proceedings of the IEEE*.

[9] Zhao S Named entity recognition in biomedical texts using an HMM model.

[10] Mccallum A, Freitag D, Pereira F Maximum entropy markov models for information extraction and segmentation.

[11] Finkel J, Dingare S, Nguyen H, Nissim M, Manning C, Sinclair G Exploiting context for biomedical entity recognition: from syntax to the web.

[12] Lafferty JD, McCallum A, Pereira FCN Conditional random fields: probabilistic models for segmenting and labeling sequence data.

[13] Settles B Biomedical named entity recognition using conditional random fields and rich feature sets.

[14] Burges CJC (1998). A tutorial on support vector machines for pattern recognition. *Data Mining and Knowledge Discovery*.

[15] Si L, Kanungo T, Huang X Boosting performance of bio-entity recognition by combining results from multiple systems.

[16] Ponomareva N, Rosso P, Pla F, Molina A (2007). *Conditional Random Fields Vs. Hidden Markov Models in a Biomedical Named Entity Recognition Task*.

[17] Liu F, Chen Y, Manderick B, Filipe J, Cordeiro J, Cardoso J (2009). Named entity recognition in biomedical literature: a comparison of support vector machines and conditional random fields. *Enterprise Information Systems*.

[18] Liu H, Hu Z-Z, Zhang J, Wu C (2006). BioThesaurus: a web-based thesaurus of protein and gene names. *Bioinformatics*.

[19] Bodenreider O (2004). The Unified Medical Language System (UMLS): integrating biomedical terminology. *Nucleic Acids Research*.

[20] Turian J, Ratinov L, Bengio Y Word representations: a simple and general method for semi-supervised learning.

[21] Kuksa PP, Qi Y Semi-supervised bio-named entity recognition with word-codebook learning.

[22] Lund K, Burgess C (1996). Producing high-dimensional semantic spaces from lexical co-occurrence. *Behavior Research Methods, Instruments, and Computers*.

[23] Hofmann T Probabilistic latent semantic analysis.

[24] Blei DM, Ng AY, Jordan MI (2003). Latent Dirichlet allocation. *Journal of Machine Learning Research*.

[25] Kanerva P, Kristoferson J, Holst A Random indexing of text samples for latent semantic analysis.

[26] Hardoon DR, Szedmak S, Szedmak O, Shawe-taylor J (2007). *Canonical Correlation Analysis; An Overview with Application to Learning Methods*.

[27] Brown PF, deSouza PV, Mercer RL, Pietra VJD, Lai JC (1992). Class-based n-gram models of natural language. *Computational Linguistics*.

[28] Bengio Y, Ducharme R, Vincent P, Jauvin C (2003). A neural probabilistic language model. *Journal of Machine Learning Research*.

[29] Bengio Y, Schwenk H, Senécal J-S, Morin F, Gauvain J-L, Holmes PDE, Jain PLC (2006). Neural probabilistic language models. *Innovations in Machine Learning*.

[30] Mikolov T, Karafiát M, Burget L, Jan C, Khudanpur S Recurrent neural network based language model.

[31] Collobert R, Weston J, Bottou L, Karlen M, Kavukcuoglu K, Kuksa P (2011). Natural language processing (almost) from scratch. *Journal of Machine Learning Research*.

[32] Mikolov T, Chen K, Corrado G, Dean J Efficient estimation of word representations in vector space.

[33] Tang B, Cao H, Wu Y, Jiang M, Xu H Clinical entity recognition using structural support vector machines with rich features.

[34] Tang B, Cao H, Wu Y, Jiang M, Xu H (2013). Recognizing clinical entities in hospital discharge summaries using Structural Support Vector Machines with word representation features. *BMC Medical Informatics and Decision Making*.

[35] Tang B, Wu Y, Jiang M, Chen Y, Denny JC, Xu H (2013). A hybrid system for temporal information extraction from clinical text. *Journal of the American Medical Informatics Association*.

[36] Ando RK BioCreative II gene mention tagging system at IBM watson.

[37] Ganchev K, Crammer K, Pereira F Penn/UMass/CHOP Biocreative II systems.

